# Causal pathways linking polycystic ovary syndrome to distinct breast cancer subtypes through mediator factors: a multivariable mendelian randomization analysis

**DOI:** 10.1186/s13048-023-01306-y

**Published:** 2023-11-13

**Authors:** Runxiang Cao, Lanlan Chen, Yutong Liu, Xuyutian Wang, Ruolin Ma, Qian Zhao, Ye Du

**Affiliations:** 1 Breast Surgery Department, General Surgery Center, First Hospital of Jilin University, Changchun, Jilin, China; 2 Hepatobiliary and Pancreatic Surgery Department, General Surgery Center, First Hospital of Jilin University, Changchun, Jilin, China

**Keywords:** Polycystic ovary syndrome, Estrogen receptor-positive breast cancer, Multivariable mendelian randomization analysis, Age at menopause

## Abstract

Polycystic ovary syndrome (PCOS) is an endocrine disorder characterized by ovarian cysts, high androgen levels, and irregular menstruation. The causality between PCOS and breast cancer (BC) has been widely discussed as they share a significant intersection in clinical manifestations. Previous epidemiological studies have not provided consistent conclusions in association between PCOS and BC, while mendelian randomization (MR) analyses have confirmed the causality between PCOS and estrogen receptor-positive breast cancer (ER + BC), but among a series of clinical manifestations resulting from PCOS, which related traits mediate the causal effect remains unknown. In this study, we conducted multivariable mendelian randomization (MVMR) analysis to explore the potential mediator variables in the mechanism linking PCOS to distinct subtypes of BC, and calculated the mediating effects proportion. We analyzed 13 PCOS-related traits and found that age at menopause may mediate PCOS-induced ER + BC (with -4.82% proportion) with a weak protective effect through the repair of DNA double-strand breaks by homologous recombination. This study helps to better comprehend the shared mechanisms contributing to the development of both PCOS and BC, and to screen high-risk populations for BC and take appropriate preventive measures.

**Supplementary Information:**

The online version contains supplementary material available at 10.1186/s13048-023-01306-y.

## Introduction

PCOS is a prevalent endocrine and metabolic disorder among women of reproductive age. Chronic anovulation and hyperandrogenism are hallmarks of PCOS, leading to clinical features such as amenorrhea, obesity, infertility, and hirsutism, which are also considered to be risk factors for BC development. The causality between PCOS and BC remains controversial, with conflicting results from previous epidemiological studies. While a Danish cohort study suggests that PCOS may increase postmenopausal BC risk [1], another retrospective cohort study indicates that it may elevate the risk of BC generally [2]. In contrast, a Taiwan cohort study has found no significant causality between PCOS and BC [3], and the largest and most recent meta-analysis conducted to date has produced consistent results [4]. In studies that have concluded PCOS leads to an increased risk of developing BC, the exact mechanisms involved are still unclear. Thus far, there have been no large-scale prospective longitudinal cohort studies carried out to investigate this issue.

The rapid development of genetics has led to the emergence of mendelian randomization (MR) in medical research. For one thing, MR studies can produce more reliable results than observational studies. First, MR follows Mendel's second law of inheritance, which states that alleles are randomly assigned at conception, avoiding the impact of residual confounding factors and reverse causation for being affected by other factors in the disease process. Second, MR can provide the impact of lifetime exposure of risk factors on outcomes. Third, genetic variants are measured with sufficient precision to avoid bias due to measurement error. For another, MR and randomized controlled trial (RCT) work on similar principles. High-quality RCTs represent the highest level of evidence. However, RCTs are often quite difficult and expensive to perform due to harsh implementation conditions and possible ethical issues. Therefore, when observational studies fail to come a consistent conclusion and RCTs are not feasible, MR may be the best method to explore causal relationships.

Two-sample MR was conducted to estimate causal relationships between one exposure and one outcome from two different samples. Estrogen receptor-positive breast cancer (ER + BC) is a hormone-dependent tumor, whereas PCOS, as an endocrine disruption disease, has proven by previous two-sample MR studies that PCOS may be a factor in the development of BC, particularly in ER + BC [5–7]. While in situations where multiple exposures are likely to be present and interact with each other, multivariable MR (MVMR) may be a more useful tool for investigating complex relationships, and can provide more robust and informative results than traditional two-sample MR. The core of MVMR relies on the fact that some genetic variants correlate more strongly with some exposures than others. MVMR pay more attention to the respective effect of each exposure in the overall effect, and correct the true causal relationship through the competition between each exposure.

We selected a series of biological markers describing the clinical manifestation of PCOS, including sex hormone binding globulin (SHBG), testosterone levels, estrogen receptor (ER) and anti-Müllerian hormone (AMH) to evaluate endocrine disorder, infertility, anovulation, age at menarche and menopause to evaluate ovarian dysfunction, insulin-like growth factor-1 (IGF-1), homeostatic model assessment for insulin resistance (HOMA-IR), triglycerides and type 2 diabetes to evaluate insulin resistance. We hypothesize that some PCOS-related traits play a mediating role in the pathway. The aim of this study is firstly to examine the independent causal associations between PCOS and distinct subtypes of BC using two-sample MR, and secondly to assess the intermediary impacts of adjustable PCOS-related risk factors in the pathogenesis of BC using MVMR. By identifying these traits and inquiring the underlying mechanisms, this study could shed light on the etiology of PCOS and BC, providing insights into prevention and intervention strategies.

## Methods

### Study design

**Fig. 1:**
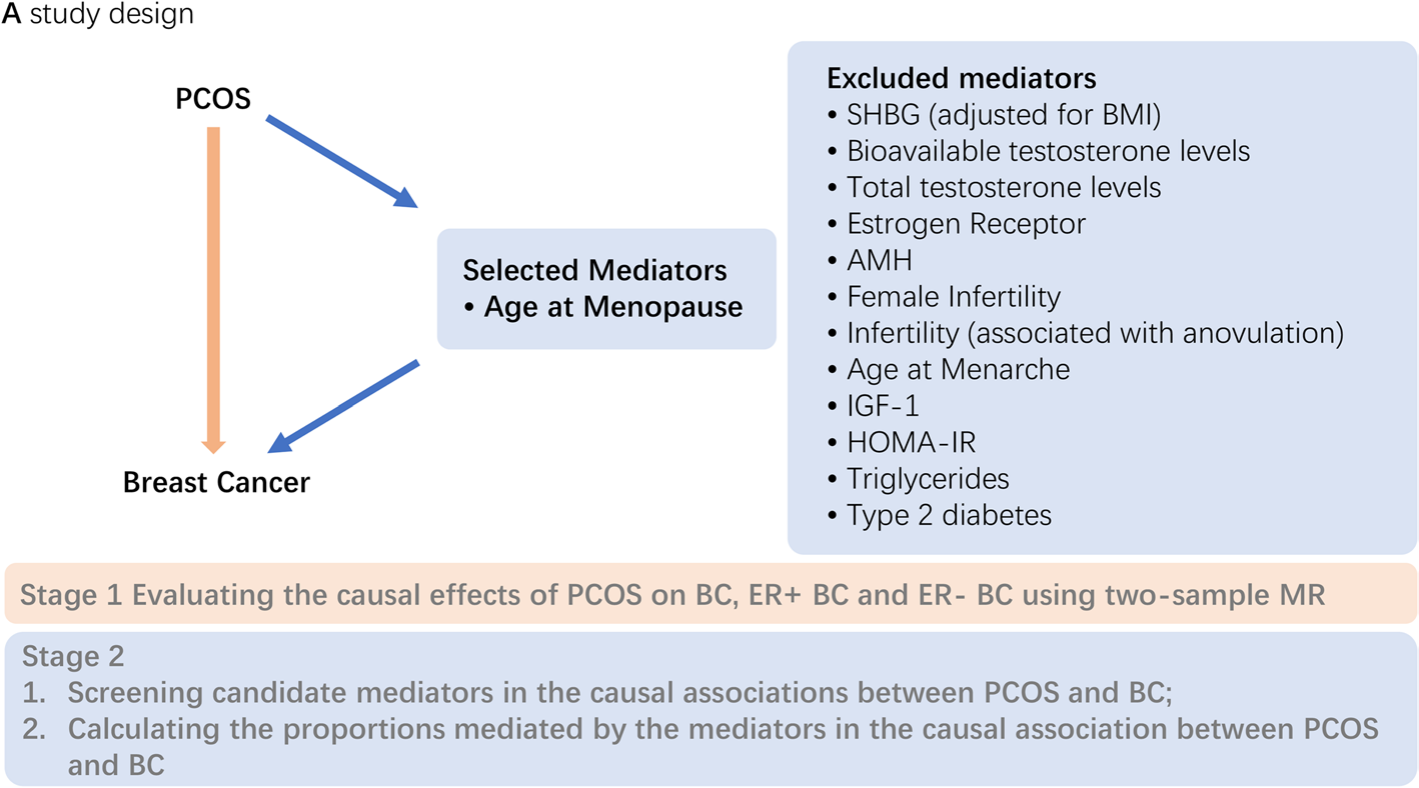
Outline of the study design. SHBG, sex hormone binding globulin; BMI, body mass index; AMH, anti-Müllerian hormone; IGF-1, insulin-like growth factor 1; HOMA-IR, homeostasis model assessment of insulin resistance

The study is comprised of two stages of analyses, as illustrated in Fig. 1, which shows the overall study design. In the first stage, we evaluated the causality between PCOS and BC distinct subtypes using two-sample MR separately. During the second stage, we detected possible mediators of the correlation between PCOS and BC and computed their intermediary effects through MVMR. Additionally, we calculated the proportions of the causality between PCOS and BC that were mediated by these mediators. The study was conducted in adherence to the STROBE-MR guidelines [8].

### Data sources

Genome-wide association studies (GWASs) summary-level data which were predominantly carried out on individuals of European descent, were employed in this study to obtain data resources for exposures, mediators, and outcomes.

#### Exposures

As the main genetic tools for PCOS, we selected 14 SNPs that were derived from the latest and largest GWAS meta-analysis of 10,074 PCOS cases and 103,164 controls with European ancestry. The meta-analysis identified 3 novel loci, as well as replicating 11 previously reported loci in Chinese and European subjects [9]. According to the rule of thumb, if the F-statistic is greater than the critical value of 10% bias in the Stock-Yogo weak ID test, the original hypothesis can be rejected and the weak instrumental variable is considered to be absent [10]. Based on this, we assessed weak instrument bias using F statistics (F > 10) to ensure the validity of the selected SNPs. F statistics was calculated using the formula: $F=\frac{{R}^{2}\left(N-2\right)}{\left(1-{R}^{2}\right)}$, and *R* ^2^ was calculated by: ${R}^{2}=\frac{2\times EAF\times \left(1-EAF\right)\times {beta}^{2}}{[(2\times EAF\times (1-EAF)\times {beta}^{2})+(2\times EAF\times (1-EAF)\times N\times SE{\left(beta\right)}^{2}}$, which is for the extended 10 SNP instruments [11]. Detailed information of the 14 independent SNPs is presented in Table 1, which gives their calculated *R* ^2^ with the F-statistics.

**Table 1:** Details of SNPs selected as exposure IVs

RSID	Effect Allele	Other Allele	EAF	beta	OR	se	*p*-Value	*R* ^2^	F
rs7563201	G	A	0.451	-0.108	0.900	0.017	3.68E-10	0.002	39.422
rs2178575	A	G	0.151	0.166	1.180	0.022	3.34E-14	0.003	57.448
rs13164856	T	C	0.729	0.124	1.130	0.019	1.45E-10	0.002	41.274
rs804279	T	A	0.262	0.128	1.140	0.018	3.76E-12	0.003	48.388
rs10739076	A	C	0.308	0.110	1.120	0.020	2.51E-08	0.002	31.175
rs7864171	A	G	0.428	-0.093	0.910	0.017	2.95E-08	0.002	30.641
rs9696009	A	G	0.068	0.202	1.220	0.031	7.96E-11	0.002	42.182
rs11031005	T	C	0.854	-0.159	0.850	0.022	8.66E-13	0.003	50.832
rs11225154	A	G	0.094	0.179	1.200	0.027	5.44E-11	0.003	43.303
rs1784692	A	G	0.824	0.144	1.150	0.023	1.88E-10	0.002	40.594
rs2271194	T	A	0.416	0.097	1.100	0.017	4.57E-09	0.002	34.141
rs1795379	T	C	0.24	-0.117	0.890	0.020	1.81E-09	0.002	35.996
rs8043701	A	T	0.815	-0.127	0.880	0.021	9.61E-10	0.002	37.276
rs853854	T	A	0.499	-0.098	0.910	0.016	2.36E-09	0.002	36.143

SNP is considered to be of adequate instrument strength with F statistics > 10. *R* ^2^ is the degree to which IV explains exposure (determinant of regression equation). *EAF* effect allele frequency

#### Mediators

A clustering analysis revealed two distinct PCOS subtypes: reproductive subtype with higher luteinizing hormone (LH) and SHBG levels, metabolic subtype with higher BMI, glucose, and insulin levels [12], and another clustering analysis identified three distinct PCOS subtypes: adiposity subtype associating with BMI and waist circumference, insulin-resistance subtype associating with fasting insulin and HOMA-IR, reproductive subtype associating with SHBG [13]. Based on these findings and previously published literature, we selected 13 clinical features and treatment measures related to PCOS and categorized them into three clusters, namely endocrine traits, reproductive traits, and metabolic traits: (1) endocrine traits such as SHBG (adjusted for BMI) [14], bioavailable and total testosterone levels [15], ER, AMH [16]; (2) reproductive traits such as female infertility, infertility associated with anovulation [17], age at menarche and age at menopause [18]; and (3) metabolic traits such as IGF-1 [19], HOMA-IR [13], triglycerides [20], and type 2 diabetes [21]. Other potential mediators, such as parity, hyperandrogenemia, and in-vitro fertilization (IVF), could not be considered as the GWAS datasets for these traits were not accessible. Detailed information on the 13 candidate mediators is presented in Table 2, which describes the ethnicity and sample size of the GWAS study.

**Table 2:** Details of GWASs included in MR analyses

Trait	Consortium	Ethnicity	Sample Size	GWAS ID
SHBG (adjusted for BMI)	EBI	European	368929	ebi-a-GCST90012110
Bioavailable testosterone levels	EBI	European	382988	ebi-a-GCST90012104
Total testosterone levels	EBI	European	425097	ebi-a-GCST90012114
Estrogen Receptor	pQTL	European	3301	prot-a-991
AMH	NA	European	7049	GCST90104596
Female Infertility	FinnGen Biobank	European	75450	finn-b-N14-FEMALEINFERT
Infertility (associated with anovulation)	FinnGen Biobank	European	118152	finn-b-N14-FIANOV
Age at Menarche	ReproGen	European	182416	ieu-a-1095
Age at menopause	ReproGen	European	69360	ieu-a-1004
IGF-1	MRC-IEU	European	468262	ukb-d-30770_irnt
HOMA-IR	MAGIC	European	37037	ieu-b-118
Triglycerides	EBI	European	94595	ebi-a-GCST002216
Type 2 diabetes	EBI	European	655666	ebi-a-GCST006867
BC(Combined Oncoarray; iCOGS; GWAS meta analysis)	BCAC	European	228951	ieu-a-1126
ER + BC(Combined Oncoarray; iCOGS; GWAS meta analysis)	BCAC	European	175475	ieu-a-1127
ER- BC(Combined Oncoarray; iCOGS; GWAS meta analysis)	BCAC	European	127442	ieu-a-1128

*EBI* European Bioinformatics Institute, *pQTL* Protein Quantitative Trait Loci Consortium, *MRC-IEU* Medical Research Council Integrative Epidemiology Unit, *FinnGen Biobank* Finnish National Genome Center Biobank, *ReproGen* Reproductive Genetics, Epigenetics & Development Group, *MAGIC* Meta-Analyses of Glucose and Insulin-related traits Consortium, *BCAC* Breast Cancer Association Consortium, *NA* not available

**Fig. 2:**
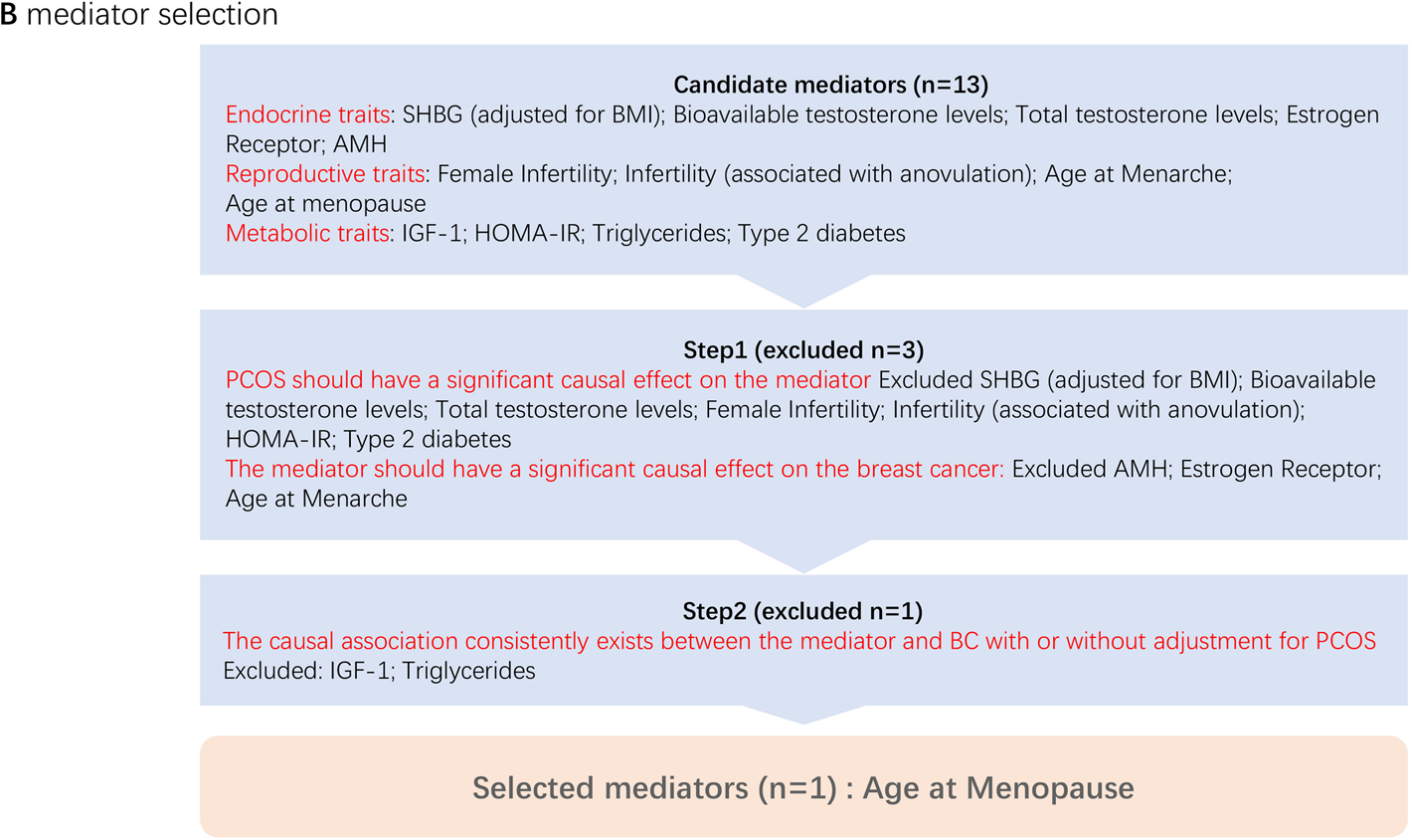
Mediator selection process

Following a rigorous selection process, our criteria for identifying potential mediators between PCOS and BC included: (1) PCOS and the mediator, as well as the mediator and BC, are causally related; (2) regardless of the adjustment for PCOS, the causality between the mediator and BC are consistent. The detailed process for selecting mediators is presented in Fig. 2, which lists the included mediators and those excluded by each step of the exclusion criteria.

Following a thorough evaluation process, age at menopause as a risk factor fulfill all selection criteria and were incorporated in the mediation analyses to assess intermediary impacts on the causality between PCOS and BC.

#### Outcomes

For genetic instruments for BC, we utilized data from a GWAS study conducted by the Breast Cancer Association Consortium (BCAC). The study comprised a sample size of 122,977 cases and 105,974 controls with European ancestry, along with 14,068 cases and 13,104 controls with East Asian ancestry, and combined data from OncoArray, iCOGS, and GWAS meta-analysis for BC, ER + BC, and ER- BC [22].

### Statistical analysis

#### Two sample MR and MVMR analyses

**Fig. 3:**
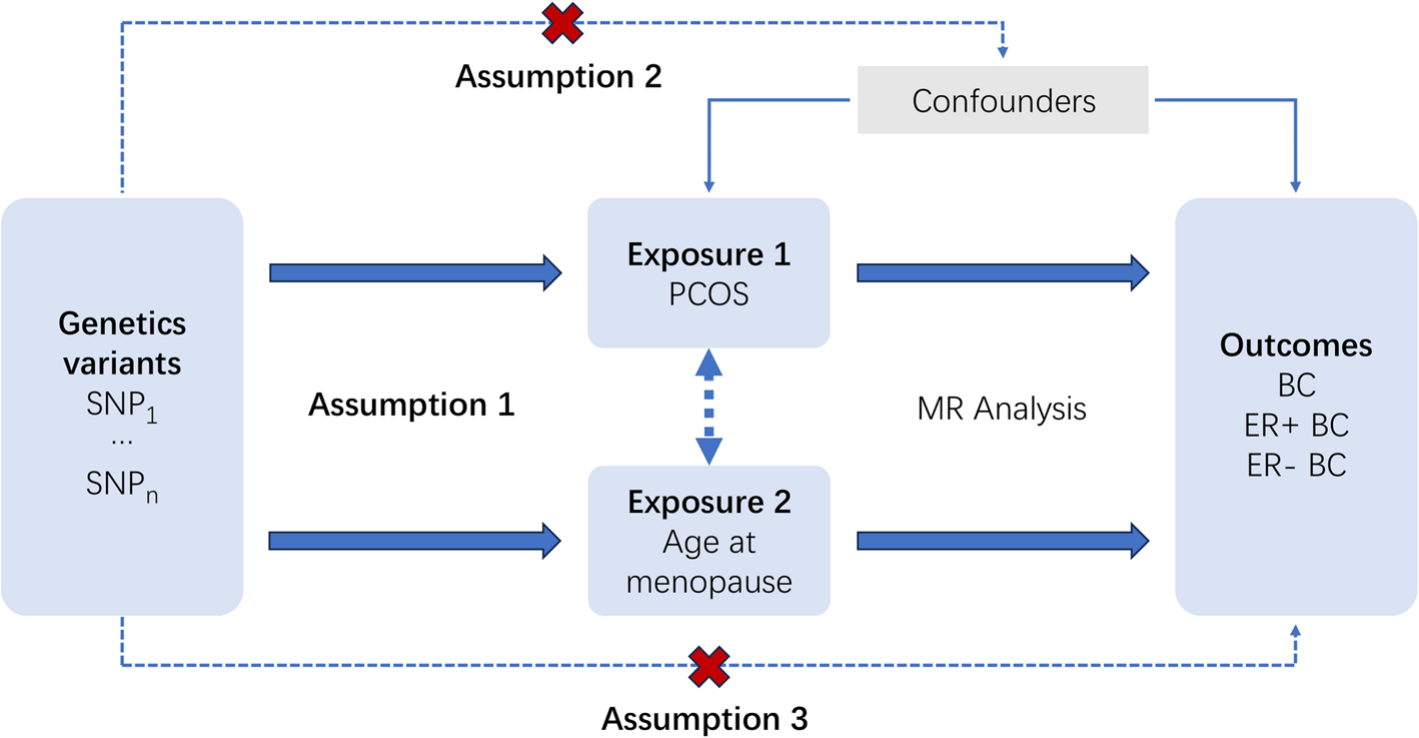
Outline of three major assumptions of MVMR

IVs for MR analyses were selected by SNPs reaching genome-wide significance (*P* < 5 × 10 − 8) and removing SNPs in linkage disequilibrium (r 2 < 0.001 or distance > 10 000 kb). All MR analyses satisfied 3 critical assumptions (Fig. 3 depicts the outline of three major assumptions of MVMR): (1) IVs must have strong correlation with the exposure in two sample MR analyses and with at least one of the multiple exposures in MVMR analyses; (2) IVs should solely influence the outcome by exerting its impact on the exposure; (3) IVs should be unaffected by any confounding variables that may influence both the exposure and the outcome variables. MVMR analysis requires the complete GWAS result to extract the IVs information. We used IVW as the primary method, which used a random-effects meta-analysis to combine the Wald Ratio estimates for each SNP into one causal estimate for each exposure. MR analyses were executed utilizing R programs "TwoSampleMR [23]", "MendelianRandomization [24]" and "MR-PRESSO [25]" in R software (version 4.2.2).

#### Mediation MR analyses

To evaluate the intermediary effects of risk factors between PCOS and BC, we conducted a mediation MR analysis. Firstly, we utilized two-sample MR to calculate the causal impact of genetically determined PCOS on the mediator (β1). Secondly, we employed MVMR to estimate the causal effect of the mediator on BC with adjustment for PCOS (β2). Finally, the indirect effect was calculated by multiplying the results from the two steps (β1 × β2) and dividing by the total effect (β). We derived standard errors by utilizing the Delta method which relied on the effect estimates generated from mediation MR analyses.

#### Sensitivity analyses

In MVMR, the direct effect of a risk factor on a disease outcome, independent of a mediator, can be estimated without being affected by any bidirectional relationship between the risk factor and mediator. This is because MVMR accounts for the bidirectional causal effects between the risk factor and mediator, and the direct effect is estimated based on the IVs that capture the genetic variation of the risk factor and mediator [26]. Therefore, the direct effect of PCOS on BC risk not via mediators can be estimated using MVMR whether or not there is a bidirectional relationship between PCOS and mediators.

## Results

### Effects of PCOS on BC

Our analyses revealed a positive association between genetically predicted PCOS and an increased risk of BC, for each 1-SD higher risk of PCOS was associated with a 6.7% elevation in overall BC risk observed using the IVW-multiplicative random effects method [odds ratio (OR) 1.067 (95% confidence interval (CI): 1.020–1.115), *p* = 0.005]. Furthermore, genetically predicted each 1-SD higher risk of PCOS was associated with an 8.8% increase in the risk of ER + BC [IVW: OR 1.088 (95% CI: 1.032–1.147), *p* = 0.002]. These findings were consistent across all methods except for MR-Egger. However, based on the IVW assessment, no significant association was observed between genetically predicted PCOS and the risk of ER- BC. The forest plot of the results is presented in Fig. 5, which illustrate the causal relationship between PCOS and BC. While some heterogeneity was observed, no evidence of pleiotropy was detected. All the Two Sample MR results are presented in Supplementary Material Table S1.

### Effects of PCOS on mediators

**Fig. 4:**
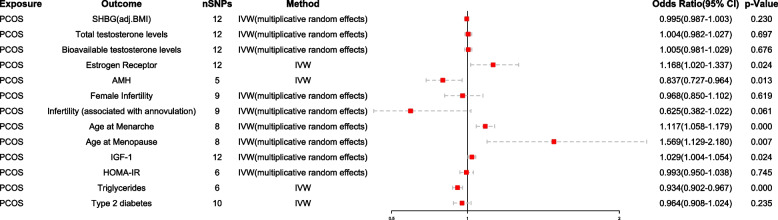
Causal effect of exposure on mediators

We observed that genetically predicted each 1-SD higher risk of PCOS was positively associated with an increased risk of ER by 16.8% [IVW: OR 1.168 (95% CI: 1.020–1.337), *p* = 0.024], age at menarche by 11.7% [IVW-multiplicative random effects: OR 1.117 (95% CI: 1.058–1.179), *p* < 0.001], age at menopause by 56.9% [IVW-multiplicative random effects: OR 1.569 (95% CI: 1.129–2.180), *p* = 0.007], and IGF-1 by 2.9% [IVW-multiplicative random effects: OR 1.029 (95% CI: 1.004–1.154), *p* = 0.024]. Conversely, genetically predicted each 1-SD higher risk of PCOS was associated with a 16.3% reduction in AMH [IVW: OR 0.837 (95% CI: 0.727–0.964), *p* = 0.013] and a 6.6% reduction in risk for high triglycerides [IVW: OR 0.934 (95% CI: 0.902–0.967), *p* < 0.001]. In contrast, there was no significant association observed between genetically predicted PCOS and SHBG (adjusted for BMI), bioavailable and total testosterone levels, female infertility, infertility associated with anovulation, HOMA-IR, or type 2 diabetes risk. The forest plot of the results is presented in Fig. 4, which visualizes the significant causal relationship between PCOS and mediators. While some heterogeneity was observed, there was no evidence of pleiotropy.

### Effects of mediators on BC

**Fig. 5:**
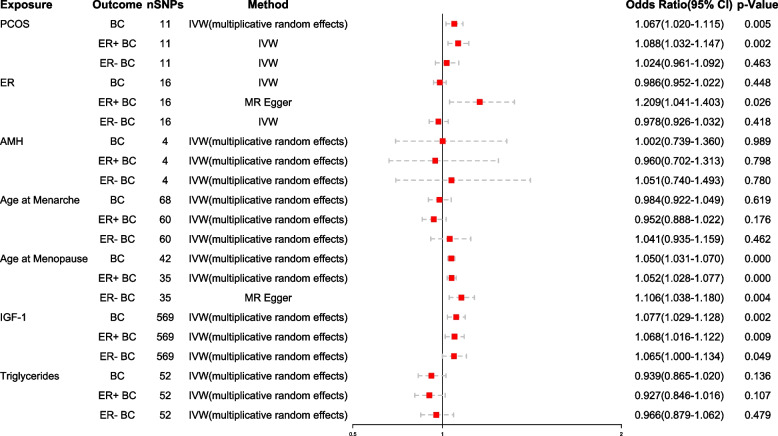
Causal effect of exposure and mediators on outcome

Our analysis revealed that genetically predicted each 1-SD higher risk of ER was positively associated with a 20.9% increased risk of ER + BC [MR-Egger: OR 1.209 (95% CI: 1.041–1.403), *p *= 0.026]. Genetically predicted each 1-SD higher risk of age at menopause was associated with a 5.0% increased risk of BC [IVW-multiplicative random effects: OR 1.050 (95% CI: 1.031–1.070), *p* < 0.001], a 5.2% increased risk of ER + BC [IVW-multiplicative random effects: OR 1.052 (95% CI: 1.028–1.077), *p* < 0.001], and a 10.6% increased risk of ER- BC [MR-Egger: OR 1.106 (95% CI: 1.038–1.180), *p* = 0.004]. Genetically predicted each 1-SD higher risk of IGF-1 was associated with a 7.7% increased risk of BC [IVW-multiplicative random effects: OR 1.077 (95% CI: 1.029–1.128), *p* = 0.002], a 6.8% increased risk of ER + BC [IVW-multiplicative random effects: OR 1.068 (95% CI: 1.016–1.122), *p* = 0.009], and a 6.5% increased risk of ER- BC [IVW-multiplicative random effects: OR 1.065 (95% CI: 1.000–1.134), *p* = 0.049]. The forest plot of the results is presented in Fig. 5, which depicts the significant causal relationship between mediators and BC.

### Mediating Effects of Mediators Between PCOS and BC

In the MVMR analysis of the relationship between PCOS, age at menopause, and ER + BC, after accounting for PCOS, the raw effect of age at menopause (where 1-SD was 3.93 years later at menopause) was negatively associated with a 7.7% decreased risk of ER + BC [IVW: OR 0.923 (95% CI: 0.861–0.989), *p* = 0.022]. The MR-PRESSO method results were consistent, and there was no evidence of pleiotropy. The proportion of the effect mediated by age at menopause was -4.82%.

**Fig. 6:**
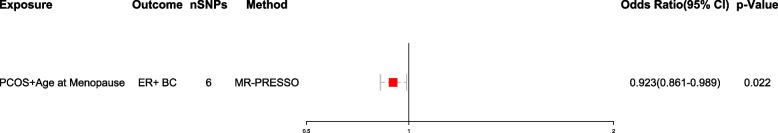
Causal effect of exposure and mediators on outcome using MVMR after outlier test

**Fig. 7:**
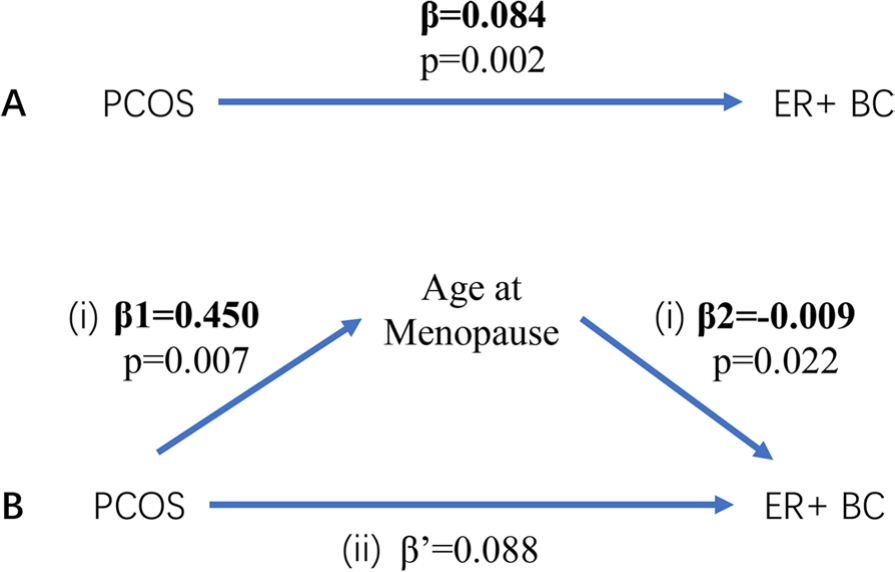
Illustrations Depicting Investigated Associations in the Study. **A** Estimation of the total effect of PCOS on BC, β, through two-sample MR. **B** Decomposition of the total effect into: (i) indirect effect, calculated using a two-step approach, in which β1 represents the total effect of PCOS on BC and β2 represents the effect of age at menopause on BC while adjusting for PCOS. (ii) direct effect (β’ = β-β1 × β2). Mediation effects were obtained by dividing the indirect effect (β1 × β2) by the total effect (β)

The forest plot of the MR-PRESSO results is presented in Fig. 6, which shows the weak protective causal effect to ER + BC after the adjustment of age at menopause to PCOS. The mediation MR analyses are presented in Fig. 7, which illustrates the direct and indirect effect with stepwise tests whether the effect is significant, and subsequently how mediation effect is calculated. The MVMR analysis of the exposure and mediators on outcome are presented in Table 3, demonstrating that age at menopause mediates the risk of PCOS-induced ER + BC after sensitivity analyses according to MRPRESSO results and excluding the remaining mediators that were not significant. All the Multivariable MR results are presented in Supplementary Material Table S2.

**Table 3:** MVMR analysis of the exposure and mediators on outcome

Exposure/Outcome	Adjusted factors	Multivariate MR analysis						Mediation effects(%)
		**Raw estimates**			**Outliers corrected estimates**			
		**N**	**Beta**	***p*** **-Value**	**N**	**Beta**	***p*** **-Value**	
PCOS/BC	Age at Menopause	6	-0.054	0.092	5	-0.005	0.683	
PCOS/ER + BC		6	-0.009	0.022	NA	NA	NA	-4.820
PCOS/ER- BC		6	-0.004	0.910	NA	NA	NA	
PCOS/BC	IGF-1	530	-0.008	0.409	501	-0.006	0.432	
PCOS/ER + BC		530	-0.005	0.630	505	-0.007	0.420	
PCOS/ER- BC		530	-0.007	0.326	522	-0.011	0.371	
PCOS/BC	Triglycerides	44	-0.009	0.768	40	-0.019	0.470	
PCOS/ER + BC		44	-0.030	0.398	38	-0.048	0.095	
PCOS/ER- BC		44	-0.046	0.360	NA	NA	NA	

The outlier test of MR-PRESSO suggested that no significant outlier was found in the causal effect between PCOS and ER + BC/ER- BC (adjusted for age at menopause), and the causal effect between PCOS and ER- BC (adjusted for triglycerides), so the outliers corrected estimates represent as NA (not available)

### Sensitivity analyses

We employed different methods depending on the presence or absence of heterogeneity and pleiotropy. Specifically, we used the inverse variance weighted (IVW) method as the main analysis when there was no heterogeneity and pleiotropy. When heterogeneity was present while pleiotropy was absent, we used the IVW-multiplicative random effects as the main analysis. When pleiotropy was detected, we employed the MR-Egger method as the main analysis and utilized the MR-PRESSO outlier test to correct for potential bias due to pleiotropy.

The results of sensitivity analyses are presented in Table 4. We selected appropriate analytical methods to ensure the robustness and reliability of the results.

**Table 4:** Heterogeneity and pleiotropy analysis

Exposure	Outcome	Heterogenity			Pleiotropy	
		**Method**	**Cochran’s Q**	***p*** **-Value**	**Egger-Intercept**	***p*** **-Value**
PCOS	Bioavailable testosterone levels	IVW	52.663	2.07E-07	-0.008	0.355
PCOS	Total testosterone levels	IVW	111.276	1.02E-18	-0.005	0.502
PCOS	AMH	IVW	6.169	1.87E-01	0.056	0.427
PCOS	Estrogn Receptor	IVW	5.503	9.04E-01	-0.039	0.404
PCOS	Female Infertility	IVW	18.558	1.74E-02	0.044	0.307
PCOS	Infertility (associated with anovulation)	IVW	53.374	9.13E-09	0.191	0.224
PCOS	Age at Menarche	IVW	14.426	4.41E-02	0.004	0.834
PCOS	Age at menopause	IVW	75.338	1.17E-11	-0.055	0.667
PCOS	IGF-1	IVW	41.795	1.76E-05	-0.013	0.114
PCOS	HOMA-IR	IVW	11.825	3.73E-02	0.006	0.783
PCOS	Triglycerides	IVW	5.089	4.05E-01	0.000	0.992
PCOS	Type 2 diabetes	IVW	15.183	8.60E-02	-0.009	0.692
PCOS	BC	IVW	48.100	3.41E-08	0.003	0.859
PCOS	ER + BC	IVW	16.200	9.40E-02	0.000	0.989
PCOS	ER- BC	IVW	3.220	9.76E-01	0.020	0.349
ER	BC	IVW	22.652	9.18E-02	-0.018	0.086
	ER + BC	MR Egger	20.633	1.11E-01	-0.034	0.018
	ER- BC	IVW	15.813	3.95E-01	-0.006	0.712
AMH	BC	IVW	52.702	2.12E-11	-0.035	0.482
	ER + BC	IVW	38.885	1.84E-08	-0.033	0.532
	ER- BC	IVW	20.650	1.24E-04	-0.026	0.669
Age at Menarche	BC	IVW	210.967	8.36E-17	0.009	0.128
	ER + BC	IVW	140.193	1.47E-08	0.012	0.065
	ER- BC	IVW	138.685	2.31E-08	0.000	0.979
Age at menopause	BC	IVW	116.165	4.11E-09	-0.005	0.243
	ER + BC	IVW	99.467	2.45E-08	-0.002	0.696
	ER- BC	MR Egger	61.421	1.92E-03	-0.015	0.024
IGF-1	BC	IVW	1764.000	1.18E-122	0.000	0.989
	ER + BC	IVW	1428.000	1.46E-75	0.000	0.838
	ER- BC	IVW	988.000	3.95E-25	-0.001	0.679
Triglycerides	BC	IVW	220.583	5.77E-23	0.005	0.127
	ER + BC	IVW	190.997	4.71E-18	0.005	0.175
	ER- BC	IVW	87.466	1.12E-03	0.002	0.557

The Cochran Q statistic was computed to analyze the degree of heterogeneity which was considered significant when *p* < 0.05, p and the MR-Egger regression was utilized to analyze the degree of pleiotropy which was considered significant when *p* < 0.05

## Discussion

In this study, we have reaffirmed the causal relationship between PCOS and BC, particularly in ER + BC. Further, our research has found one mediator out of 13 PCOS-related traits, age at menopause, with mediating proportion of -4.82% in the association between PCOS and ER + BC.

One of the causes of endocrine disruption in PCOS is abnormal regulatory function of the hypothalamic-pituitary-ovarian (HPO) axis. Due to the increased sensitivity of the pituitary gland to gonadotropin-releasing hormone (GnRH), it secretes excessive LH, stimulating the cells of the ovarian mesenchyme and follicular membrane to produce excessive androgens. Hyperandrogenism in the ovary inhibits follicular maturation and prevents the dominant follicles formation, but small follicles can still secrete estradiol equivalent to the early follicular phase levels, coupled with the conversion of androstenedione to estrone under the impact of aromatase in peripheral tissues, generating the formation of hyperestrogenemia. Therefore, patients with PCOS not only have excess androgen levels, but also have high estrogen levels. Continuous secretion of estrone and a certain level of estradiol acts on the pituitary gland and hypothalamus, and positively feedbacks on LH secretion, resulting the amplitude and frequency of LH secretion increase with a sustained high level of no cyclicity, and no formation of mid-menstrual LH peaks, so no ovulation occurs. Estrogen in turn exerts negative feedback on follicle-stimulating hormone (FSH) secretion, resulting a relative decrease in FSH levels and an increase in the LH/FSH ratio. High levels of LH boost the secretion of androgens, while low levels of FSH stop the small follicles development, with no dominant follicle formed, thus starting up a vicious cycle of excessive androgens and continuous anovulation, contributing to polycystic changes in the ovary.

Androgens, as precursors to estrogens, can contribute to excessive estrogen production and subsequent breast cell proliferation when present in excess. Testosterone is converted into two antagonistic metabolites: estradiol binds to estrogen receptor to stimulate breast epithelial cell proliferation, while dihydrotestosterone binds to androgen receptor to inhibit this process. At the beginning of hormone-dependent tumor growth, the levels of both hormones are higher than in normal cells, and their effects are counterbalanced. However, if excess androgen levels are not eliminated, the proliferative effects of estrogen will ultimately prevail. Excess androgens can also result in elevated production of epidermal growth factor, a recognized stimulant of breast epithelial cell proliferation. Several prospective studies conducted in postmenopausal females have demonstrated a correlation between elevated circulating androgen levels and serum testosterone levels with an increased susceptibility to breast cancer [27–29]. PCOS is linked to significantly elevated AMH levels compared to those found in normally ovulating women. The identification of a correlation between elevated circulating levels of AMH and the occurrence of BC lends support to the feasibility of utilizing AMH as a biomarker for the detection of BC [30].

Another pathogenesis of metabolic disorder of PCOS is insulin resistance and hyperinsulinemia. Reduced sensitivity of peripheral tissues to insulin and lower than normal biological efficacy of insulin is known as insulin resistance. About 50% of patients have varying degrees of insulin resistance and compensatory hyperinsulinemia. Excess insulin acts on insulin receptors in the pituitary gland to enhance LH release and promote androgen secretion from the ovaries and adrenal glands, which in turn increases free testosterone by inhibiting hepatic SHBG synthesis. Elevated insulin levels may encourage cell growth and division while affecting estrogen synthesis, metabolism, and signaling pathways, with increasing IGF-1 levels, which promote tumor growth and dissemination.

The pharmacological management of PCOS mainly focuses on alleviating metabolic abnormalities, including the use of oral contraceptive pills to regulate menstrual cycles, steroids to reduce blood androgen levels, metformin to relieve insulin resistance, clomiphene and FSH to induce ovulation, and additionally, IVF to restore fertility. Ovulation-inducing medications can result in increased estradiol levels. Furthermore, clomiphene may diminish estrogen receptor activity in certain tissues and has demonstrated a direct pro-apoptotic impact on BC cell lines, indicating a possible anti-cancer effect. IVF may heighten the BC risk due to hormonal fluctuations during the IVF procedure. Additionally, IVF could lead to the development of more blood vessels in breast tissue, which might offer a route for cancer cells to disperse. In women with impaired glucose homeostasis, metformin could potentially reduce BC risk by lowering insulin levels.

In the pathway from PCOS to ER + BC, -4.82% of the mediation effect was explained by age at menopause. When both direct and indirect effect are significant, but in opposite direction, previous study has described it as suppression effect [31]. According to the results of two-sample MR, genetically predicted PCOS is associated with later age at menopause, and later menopausal age is associated with higher risk of developing BC, which are both consistent with current epidemiological research perspectives [32]. However, after adjusting for the interaction between the two exposures with MVMR method, a weak protective effect against BC was detected, which is worth further investigation. One study has found age at menopause associated SNPs strongly enriched in DNA damage response (DDR) pathways [33]. As menopausal age is delayed, there is an increase of the DDR gene mutation and the BRCA1 gene expression, which promotes the repair of DNA double-strand breaks through homologous recombination. In other words, later menopausal age plays a role in tumor inhibition and BC risk reduction. This seems to be paradoxical with the epidemiological view. However, the study noted that the association between menopausal age variants and BC risk in DDR genes is weaker compared to those in non-DDR genes set, so hormone exposure duration plays a dominant role, producing a greater risk effect to offset the weak protective effect. MVMR may have corrected the interaction of the hormones effect between PCOS and age at menopause (i.e., the shared pathway between two exposures), and the calculated mediating proportion is the uncorrected effect produced by menopausal age variants in DDR pathways, playing a role as homologous recombination repair and tumor inhibition.

Given the causal impact and elevated prevalence of PCOS and BC, identification of this association could facilitate the creation of a screening program for individuals at elevated risk, as well as the implementation of primary interventions to mitigate their risk. We can suggest patients with PCOS start regular screening for breast examination, breast ultrasound, and mammography at an earlier age, instead of the guideline-recommended screening once every 6–12 months for those who are 40 years old or older. For patients found having benign breast nodules, closer follow-up is required, and core needle biopsy or vacuum assisted biopsy need to be taken into consideration more sensitively. When using endocrine medications for treatment, it is important to carefully monitor their potential interacting effects on the breast. As an aromatase inhibitor, letrozole is commonly employed in treating anovulatory infertility in PCOS. Compared to clomiphene, letrozole demonstrates better tolerability and fewer adverse effects on endometrium and cervical mucus, which can be used in patients with low response to clomiphene. Furthermore, letrozole is utilized as an adjuvant therapy in postmenopausal ER + BC patients, suggesting the possibility of a decreased risk of hormonal-dependent cancer. Considering the comparable pathogenesis underlying both PCOS and BC, common genes in the pathogenesis have been explored, which could be potential targets of treatment for both PCOS and BC [34].

More than 95% of the effect was not explained by the mediators included in the model. The null findings may be attributed to (1) the explanatory power of the instrumental variables (IVs) for the mediator is insufficient; (2) the mediator from PCOS to BC is inherently rare or has a weak mediating effect; (3) the presence of other unconsidered mediators. Our selection of mediators was limited to endocrine and metabolic pathways. This suggests we need to look for suitable mediators from other perspectives. New insights into genetic recombination and repair may be gained from future large-scale genomic studies. Consequently, we should interpret the results cautiously, and further research should delve into additional potential mediators and utilize supplementary approaches to validate the conclusions.

The study yielded an unexpected finding whereby a subset of potential mediators previously supported by robust observational studies, were found to be non-intermediary variables in the pathogenesis of BC from PCOS. Apart from unobserved confounding factors or other types of biases, null results may be elucidated by the collinearity between PCOS and the mediators. In other words, there may be an interaction between PCOS and mediators. As a whole, the effects induced by PCOS through complex endocrine and metabolic pathways encompass the effects induced by mediating factors. Therefore, when PCOS and mediators are included in the MVMR, their effects may be masked or offset, resulting in their causal relationship with BC no longer being significant. Using MVMR method, we identified possible DDR gene pathways of age at menopause other than endocrine pathways while unable to find positive results in other mediators.

The primary advantage of our study is the novel contribution of being the first MVMR study to identify causal mediators in the pathway connecting PCOS and BC. The MVMR approach has the advantage of accommodating the multiple mediators simultaneously and consider the combined impacts, even in the presence of bidirectional relationships. Meanwhile, we applied rigorous criteria to ensure the validity and plausibility of the model we constructed to explain the mediating effect.

However, we recognize certain drawbacks to our research. The absence of standardized diagnostic criteria for PCOS, including Rotterdam criteria, NIH/NICHD criteria, and self-reported diagnosis, has led to heterogeneity in the generated GWAS data sets of PCOS. Thereby, MR studies employing these data sets have not been able to detect the impacts of different phenotypes. A previous investigation has recognized reproductive and metabolic categories of PCOS, which seem to exhibit separate genetic frameworks, given that the PCOS diagnostic criteria fail to differentiate between biologically distinct disease subtypes [12]. Future research can overcome this concern when information regarding discrete subtypes of PCOS becomes accessible. Third, our BC outcome data were not stratified by menopausal status, limiting the ability to determine the effect of exposure and mediator in women of different menopausal statuses. Forth, the mediator GWAS data was composed of both genders; however, the exposure GWAS data of PCOS and outcome GWAS data of BC consisted solely of female. The MR analyses would have been more robust from a more consistent gender composition across exposure, outcome, and mediators. Lastly, the study population consisted solely of individuals of European descent, raising questions about the generalizability of the results of MR studies employing these genetic instruments to other populations. Future studies with more ethnic populations included are needed.

Since 2009, China has implemented a free public health program for BC screening, which has significantly reduced the morbidity and mortality rates of female BC. Gaining an insight into the relationship between PCOS and BC development will help public health policies to incorporate PCOS patients in the BC high-risk cohort in the future, and to spread the concept of "early prevention, early detection and early treatment" to the general population, especially target populations.

### Supplementary Information

**Additional file 1.**

## Data Availability

Summary statistics are available from the IEU OpenGWAS database (https://gwas.mrcieu.ac.uk/), GWAS: Catalog of Published Genome-Wide Association Studies, RRID:SCR_012745, (https://www.ebi.ac.uk/gwas/), and the FINNGEN database (https://r8.finngen.fi/). Original data generated and analyzed during this study are included in this published article.

## Abbreviations

BC: Breast cancer
ER + BC: Estrogen receptor-positive breast cancer
ER - BC: Estrogen receptor-negative breast cancer
GWAS: Genome-wide association study
IV: Instrumental variable
IVW: Inverse variance weighted
MR: Mendelian randomization
MR-PRESSO: Pleiotropy RESidual Sum and Outlier
MVMR: Multivariable mendelian randomization
PCOS: Polycystic ovary syndrome
SNP: Single nucleotide polymorphism
STROBE-MR: Strengthening the Reporting of Observational Research in Epidemiology using Mendelian Randomization
